# Phenonaut: multiomics data integration for phenotypic space exploration

**DOI:** 10.1093/bioinformatics/btad143

**Published:** 2023-03-21

**Authors:** Steven Shave, John C Dawson, Abdullah M Athar, Cuong Q Nguyen, Richard Kasprowicz, Neil O Carragher

**Affiliations:** Edinburgh Cancer Research, Cancer Research UK Scotland Centre, Institute of Genetics and Cancer, University of Edinburgh, Edinburgh EH4 2XR, United Kingdom; GlaxoSmithKline Medicines Research Centre, Stevenage SG1 2NY, United Kingdom; Edinburgh Cancer Research, Cancer Research UK Scotland Centre, Institute of Genetics and Cancer, University of Edinburgh, Edinburgh EH4 2XR, United Kingdom; GlaxoSmithKline Medicines Research Centre, Stevenage SG1 2NY, United Kingdom; Artificial Intelligence and Machine Learning, GlaxoSmithKline, South San Francisco, California 94080, United States; GlaxoSmithKline Medicines Research Centre, Stevenage SG1 2NY, United Kingdom; Edinburgh Cancer Research, Cancer Research UK Scotland Centre, Institute of Genetics and Cancer, University of Edinburgh, Edinburgh EH4 2XR, United Kingdom

## Abstract

**Summary:**

Data integration workflows for multiomics data take many forms across academia and industry. Efforts with limited resources often encountered in academia can easily fall short of data integration best practices for processing and combining high-content imaging, proteomics, metabolomics, and other omics data. We present Phenonaut, a Python software package designed to address the data workflow needs of migration, control, integration, and auditability in the application of literature and proprietary techniques for data source and structure agnostic workflow creation.

**Availability and implementation:**

Source code: https://github.com/CarragherLab/phenonaut, Documentation: https://carragherlab.github.io/phenonaut, PyPI package: https://pypi.org/project/phenonaut/.

## 1 Introduction

As academic drug discovery efforts further embrace multiparametric assay technologies and multiomic dataset generation (Hasin et al. 2017), the management of data integration pipelines becomes increasingly critical. This is addressed in industrial drug discovery efforts driven by compliance requirements and resource availability. Early-stage academic efforts are typically less well supported, tackling targets and diseases areas with different risk/reward profiles (Tralau-Stewart et al. 2009) and playing to the strengths of individual groups whilst affording more freedom and less oversight. This encourages *ad hoc* analysis with scientists often acting to collect, process, and interpret data with single-use workflows, lacking oversight, tracking, and validation. We present Phenonaut, a Python package for the analysis of multiomic biological data in a compliant and reproducible manner. With a focus on multiparametric multiomic data, Phenonaut differentiates itself from other available software packages, such as BIOVIA Dassault Systèmes’ Pipeline Pilot, KNIME (Berthold et al. 2009), Core Life Analytics’ StratoMineR, TIBCO Spotfire^®^, Pycytominer (Way et al. 2019), SCANPY (Wolf et al. 2018), and SnakeMake (Köster and Rahmann 2012), all of which are limited by their cost, closed nature, or focus on one specific omics technology. In the hope to revolutionize featurized multiomics workflows in the same way that SnakeMake has revolutionized bioinformatics workflows, Phenonaut addresses the following data needs; (i) Migration—use of the rich Python ecosystem allows access to many formats and protocols. (ii) Control—use of the Python API or YAML workflow mode allows automation, control, testing, and deployment in HPC and cloud environments. (iii) Integration—Phenonaut is designed to work with multiomics data, taking multiple views into an underlying biological system e.g. imaging accompanied by proteomics. (iv) Auditability; Phenonaut runs are accompanied by cryptographic hashes proving reported inputs and workflows produced certain outputs.

## 2 Implementation

**Figure 1:**
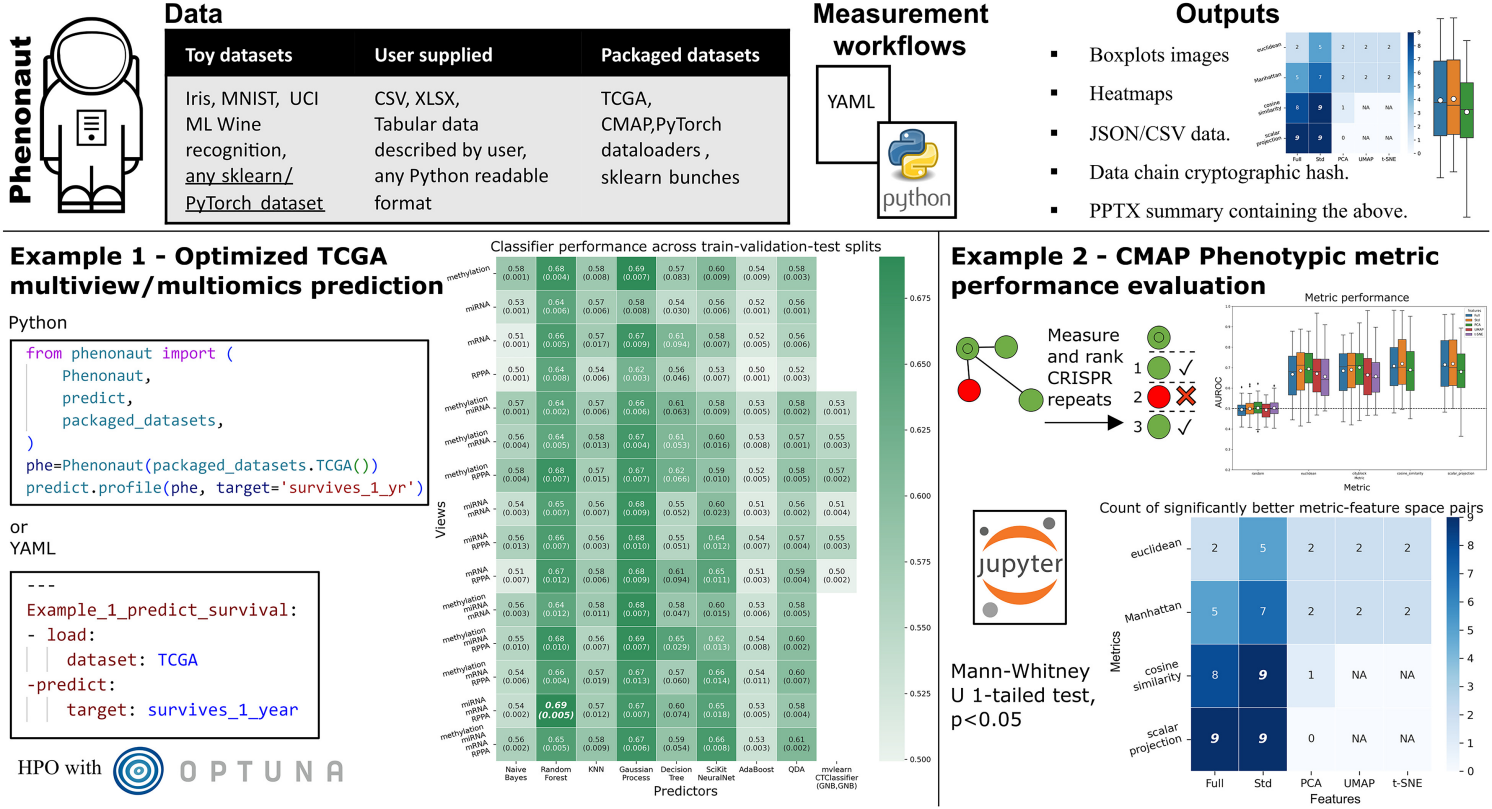
Phenonaut exemplified using its packaged dataset loaders. Lower left: Multiomics dataset combinations for the Cancer Genome Atlas are profiled with hyperparameter optimized classifiers to predict 1 year donor survival rate. Lower right: Phenotypic metrics are profiled against A549 cell line CRISPR repeats in Connectivity Map and assessed via AUROC scores to as to their repeat enrichment in ranked hit lists

Written in Python, Phenonaut is a pip-installable package for data integration and workflow generation. Users do not have to be proficient in the Python programming language, as Phenonaut implements a workflow mode using simple YAML files. See Supplementary Information for user guide with further details as well as online API documentation. Although initially developed for the analysis of phenotypic screening campaigns featurized with CellProfiler (Carpenter et al. 2006), Phenonaut is input data structure agnostic, allowing users to describe the structure of data. This flexible approach allows diverse formats to be processed and used in multiomics workflows. Multiomic capabilities are exemplified in Fig. 1, example 1 (lower left), whereby The Cancer Genome Atlas (Weinstein et al. 2013) loaded as a packaged dataset included in Phenonaut is used with the predict submodule to assess a range of machine learning techniques against all possible combinations of methylation, miRNA, mRNA, and reverse phase protein array data in predicting the one-year survival rate of tumour donors. This profiling can easily be adapted to hit calling/phenotype assignment within multiomics datasets, enabling scientists without coding experience to access and optimize state-of-the art methods. Output from this profiling process with user-defined or inbuilt metrics consists of performance heatmaps highlighting best view/predictor combinations in bold, boxplots for each view combination and a PPTX presentation file allowing easy sharing of data, along with machine-readable CSV/TSV and JSON results. A second example in Fig. 1 (lower right) showcases the use of the Connectivity Map (Lamb et al. 2006) for the evaluation of commonly used phenotypic metrics (Warchal et al. 2016). See Supplementary Information for further information on given examples.

## 3 Conclusions

With continued use, development, promotion, and community engagement, we anticipate the implementation of further novel and established literature techniques integrated into Phenonaut, as well as inviting community contributions via email or GitHub pull requests. We envision Phenonaut becoming a gold standard workflow integration tool for multiparametric multiomics data within the fields of phenotypic screening, biomarker discovery, and beyond.

## Supplementary Material

btad143_Supplementary_Data

## Data Availability

The data underlying this article are available in the article and in its online supplementary material.
