# Supplementary material for: Phenonaut: multiomics data integration for phenotypic space exploration
Source: Bioinformatics. 2023 Mar 21;39(4):btad143. doi: 10.1093/bioinformatics/btad143 (PMC10068743; doi:10.1093/bioinformatics/btad143)
Supplement: btad143_Supplementary_Data [file btad143_supplementary_data.docx]

Phenonaut; multiomics data integration for phenotypic space exploration

Steven Shave, John C Dawson, Abdullah M Athar, Cuong Q Nguyen, Richard Kasprowicz and Neil O Carragher

# Licencing

This is an Open Access article distributed under the terms of the Creative Com-mons Attribution License (http://creativecommons.org/licenses/by/4.0), which permits unrestricted use, distribution, and reproduction in any medium, provided the original work is properly credited. Development of material in this manuscript has been supported by GSK.

# Formatting preamble

PYTHON Code sections are in the following style:

n_squared_dict={n:n**n for n in range(1, 8)}

print(n_squared_dict)

YAML sections are in the following style:

---

Example_1_predict_survival:

- load:

    dataset: TCGA

- predict:

    target: survives_1_yr

Console/file output has the following style:

{1: 1, 2: 4, 3: 27, 4: 256, 5: 3125, 6: 46656, 7: 823543}

# Phenonaut user guide

For a full listing of capabilities, classes and functions available within Phenonaut, please refer to the online API documentation which is constantly expanding as new literature techniques are integrated.

## The Phenonaut object

A typical Phenonaut run begins with instantiation of a Phenonaut object.

Many options for instantiation are permitted, with an empty object created if no parameters are given.

from phenonaut import Phenonaut

phe=Phenonaut()

Please refer to Phenonaut API documentation for a complete description of Phenonaut constructor arguments and member functions.

An instantiated Phenonaut object has the following member variables (see Figure S1):

- datasets – a list of Dataset objects.
- df - shorthand notation to access the Pandas DataFrame of the last Dataset in the datasets list.
- ds - shorthand notation to access the last Dataset in the datasets list.
- name – string containing a name for the Phenonaut object, this simplifies working with multiple Phenonaut objects in list-like structures.


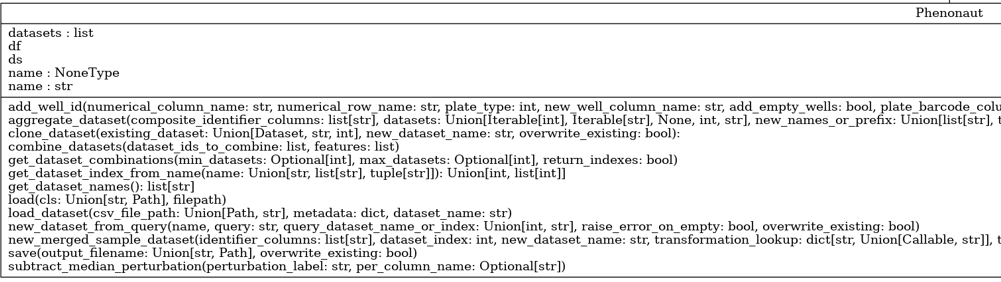


Figure S1 – Snapshot of Phenonaut object with member variables and member functions shown in the lower portion of the image. Full typing hinting is too long to display here, but available in Phenonaut API documentation (<https://carragherlab.github.io/phenonaut>).

In addition to these member variables, we can access the datasets list using dictionary-like accessors. For example, with an instantiated Phenonaut object stored in the variable “phe” containing two datasets “Alice” and “Bob”, we may access them using dictionary-like and list-like accessors.

phe["Alice"] # Access the Alice Dataset

phe["Bob"] # Access the Bob Dataset

phe[0]  # Access the first (Alice) Dataset

phe[1]  # Access the second (Bob) Dataset

phe[-1] # Access the last (Bob) Dataset

Phenonaut objects along with all data they hold may be easily written to python pickle files:

phe.save("phe.pkl")

Pickled Phenonaut objects may be loaded in the following way:

phe=Phenonaut.load("phe.pkl")

## Datasets

Datasets in Phenonaut encapsulate Pandas DataFrames and provide functionality to monitor features within the DataFrame, along with metadata, and provide a consistent interface for Phenonaut’s transforms, predictions and metrics (see Figure S2).


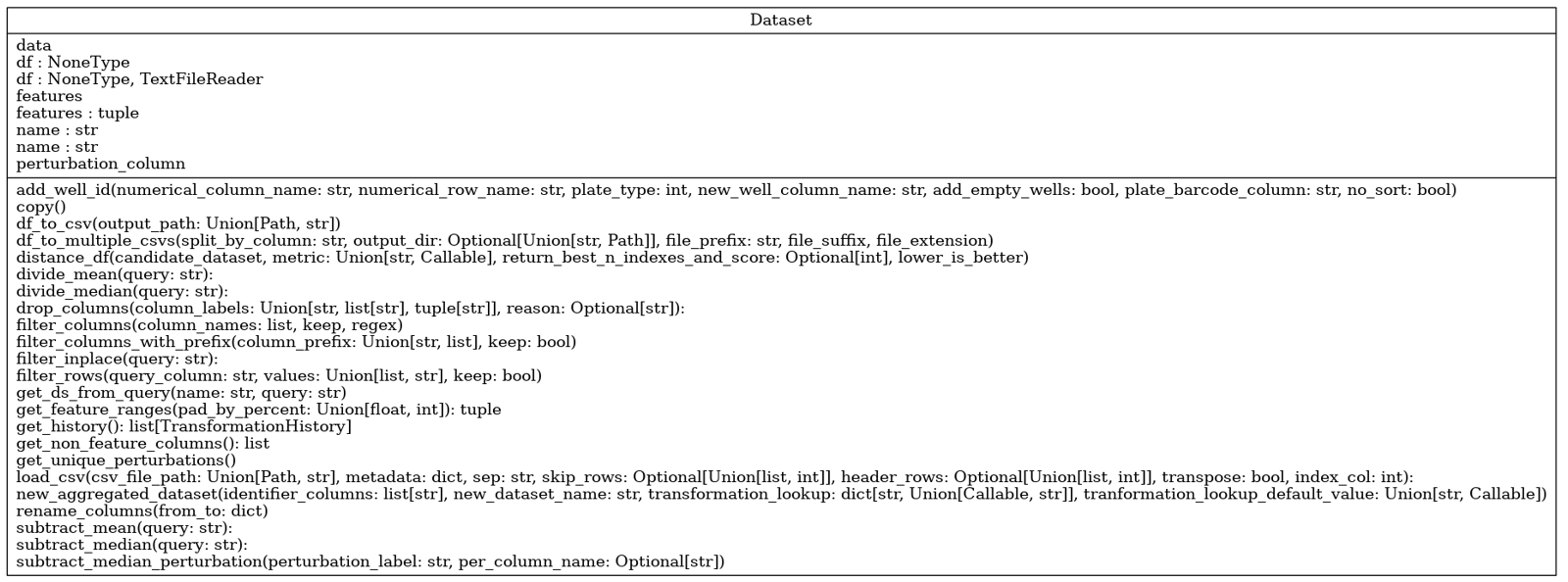


Figure S2 –Dataset object with member variables and member functions shown in the lower portion of the image.

A user supplied CSV file may be used to instantiate a Dataset object. Using a CSV file containing the following small sample of the Iris dataset:

sepal length (cm),sepal width (cm),petal length (cm),petal width (cm),target

5.4,3.4,1.7,0.2,0

7.2,3.0,5.8,1.6,2

6.4,2.8,5.6,2.1,2

4.8,3.1,1.60.2,0

5.6,2.5,3.9,1.1,1

It may be loaded into a Dataset object using three different feature selection methods:

1. The ‘features’ key, specifying a list of features.
2. The ‘features_prefix’ key, specifying a common prefix.
3. The ‘features_regex’ key, specifying a regular expression.

All three feature selection methods/keys may be used in combination with the others, with the final list of selected features across methods being uniquified and sorted.

Feature selection using the ‘features’ metadata key:

from phenonaut.data import Dataset

ds = Dataset(

    "small iris",

    "projects/iris/small_iris.csv",

    {"features": ["sepal length (cm)", "sepal width (cm)", "petal length (cm)", "petal width (cm)"]},

)

Feature selection using the ‘features_prefix’ metadata key (note that multiple prefixes can be given in a list as shown below, specifying ‘sepal’ and ‘petal’).

from phenonaut.data import Dataset

ds = Dataset(

     "small iris",

     "projects/iris/small_iris.csv",

     {"features_prefix": ["sepal", "petal"]},

)

Feature selection using the ‘featrures_regex’ metadata key, here, we match the words ‘width’ or ‘length’ surrounded by any number of other characters.

from phenonaut.data import Dataset

ds = Dataset(

     "small iris",

     "projects/iris/small_iris.csv",

     {"features_regex":".*(width|length).*"},

)

Please refer to Phenonaut API documentation for a complete description of Dataset arguments.

The dataset object may then have its features queried:

print(ds.features)

Resulting in the output:

['petal width (cm)', 'sepal width (cm)', 'petal length (cm)', 'sepal length (cm)']

Accessing the underlying DataFrame is achieved as follows:

ds.df

A DataFrame of only features may also be accessed via the .data property, useful as input to transforms.

ds.data # equivalent to calling ds.df[ds.features]

If we wish to change the features, for example, by removing “sepal length (cm)”, we may supply the updated list of features, along with a description of the change in text format which is logged:

new_features=[f for f in ds.features if f != "sepal length (cm)"]

explanation_string="Removed sepal length (cm) feature"

ds.features=(new_features, explanation_string)

Note we set features to a tuple with the first element being a list containing the new features, and the second element being an explanation string.

The history of a dataset can be examined through accessing the ‘history’ property of the dataset, or calling .get_history() with both returning a list of named tuples representing the changes a dataset went through. For all history tuples, the first element is a list of features (also accessible through calling .features on the NamedTuple), and the second element is a history message (accessible through calling .description on the NamedTuple).

ds.history

or

ds.get_history()

Resulting the return of a list of history tuples.

[TransformationHistory(features=['petal length (cm)', 'petal width (cm)', 'sepal length (cm)', 'sepal width (cm)'], description='Loaded /tmp/tmpcu2rpsai, sep=\',\'", skiprows=None, header=[0], transpose=False, index_col=None, metadata={\'features_regex\': \'.*(width|length).*\', \'initial_features\': [\'petal length (cm)\', \'petal width (cm)\', \'sepal length (cm)\', \'sepal width (cm)\']}, init_hash=None, '), TransformationHistory(features=['petal length (cm)', 'petal width (cm)', 'sepal width (cm)'], description='Removed sepal length (cm) feature')]

Datasets can be passed to the constructors of Phenonaut objects to instantiate them with the dataset.

from phenonaut import Phenonaut

from phenonaut.data import Dataset

ds = Dataset(

    "small iris",

    "projects/iris/small_iris.csv",

    {"features": ["sepal length (cm)", "sepal width (cm)", "petal length (cm)", "petal width (cm)"]},

)

phe=Phenonaut(ds)

Alternatively, we can add a dataset to an already instantiated Phenonaut object using dictionary-like keys:

phe["new small iris"]=ds

Rather than creating a Dataset object to pass to Phenonaut, we may call the load_dataset function of an instantiated Phenonaut object to load a CSV file into a dataset.

from phenonaut import Phenonaut

phe=Phenonaut()

phe.load_dataset("small iris", "projects/iris/small_iris.csv",

    {"features": ["sepal length (cm)", "sepal width (cm)", "petal length (cm)", "petal width (cm)"]}

)

## Datasets containing one feature per-row

By default, Phenonaut expects features to be present in columns, with one column capturing a feature, and one row representing a sample, like:

Table S1 – Exemplary Phenonaut dataset, containing features in columns

| sepal length (cm) | sepal width (cm) | petal length (cm) | petal width (cm) | target |
| --- | --- | --- | --- | --- |
| 5.4 | 3.4 | 1.7 | 0.2 | 0 |
| 7.2 | 3.0 | 5.8 | 1.6 | 2 |
| 6.4 | 2.8 | 5.6 | 2.1 | 2 |
| 4.8 | 3.1 | 1.60 | 2 | 0 |
| 5.6 | 2.5 | 3.9 | 1.1 | 1 |

In the above dataset (Table S1), we may indicate to Phenonaut that the features are:

- sepal length (cm)
- sepal width (cm)
- petal length (cm)
- petal width (cm)

All other columns (in this case, ‘target’) are deemed metadata columns.

Phenonaut is data structure agnostic, instead of having dataloaders for specific formats, Phenonaut relies upon transformations at the read-in stage to manipulate different formats into the above column features-format.

Below, we see an example (fake data, often encountered structure) dataset from DRUG-seq, with just 2 ensemble IDs being measured across 4 wells (A1, A2, A3, and A4). It is vastly different to the internal format required by Phenonaut.

Table S2 - DRUG-seq sample output, 2 measurements per well, for 4 wells.

|  | **Var1** | **Var2** | **Value** |
| --- | --- | --- | --- |
| 1 | ENSG00000225972 | A1_CPD1_PLATE1 | 4.0185 |
| 2 | ENSG00000225630 | A1_CPD1_PLATE1 | 1.1539 |
| 3 | ENSG00000225972 | A2_CPD2_PLATE1 | 10.6661 |
| 4 | ENSG00000225630 | A2_CPD2_PLATE1 | 1.6130 |
| 5 | ENSG00000225972 | A3_CPD3_PLATE1 | 0.1234 |
| 6 | ENSG00000225630 | A3_CPD3_PLATE1 | 9.8436 |
| 7 | ENSG00000225972 | A4_Pos_ctrl_PLATE1 | 0.1234 |
| 8 | ENSG00000225630 | A4_Pos_ctrl_PLATE1 | 9.8436 |

Fortunately, Phenonaut offers the ability to perform transforms on the data upon reading it in. This is achieved through use of the ‘transforms’ key in metadata. The ‘transforms’ key should have under it a list of transforms to be applied to the data. These transforms can be any member function of the Phenonaut Dataset class, and has the form:

('replace_str',('target_column', 'Pos_','Pos-')),

('replace_str','target_column', 'Pos_','Pos-'),

The above shows 2 ways to invoke the phenonaut.data.Dataset.replace_str function, with list elements consisting of tuples in two forms:

- 2 elements in the tuple, 1st element being the name of the function to be called, and the second element as a tuple of arguments to be passed to that function.
- n-elements in the tuple, whereby the 1st element is the name of the function to be called, and subsequent elements are arguments to be passed to the function.

We have features listed one per-row and occurring once for every well on a plate measured.

Our target format for this dataset is shown below (Table S3):

Table S3 – Target format for example DRUG-seq data

| **WellName** | **CpdID** | **PlateID** | **ENSG00000225972** | **ENSG00000225630** |
| --- | --- | --- | --- | --- |
| A1 | CPD1 | PLATE1 | 4.0185 | 1.1539 |
| A2 | CPD2 | PLATE1 | 10.6661 | 1.6130 |
| A3 | CPD3 | PLATE1 | 0.1234 | 9.8436 |
| A4 | Pos_ctrl | PLATE1 | 0.1234 | 9.8436 |

To achieve this we need to carry out transforms when reading in the csv file. We need to address the following with transforms:

- The ‘Var2’ column contains underscores which we may split on to generate new columns containing the WellName, CpdID and PlateIDs. however, the last two lines of the file contains a different number of underscores in the Var2 column than all other wells. This is due to the presence of: ‘A4_Pos_ctrl_PLATE1’. We must therefore replace the ‘Pos_’ text with a character that we will not split on.

("replace_str", "Var2", "Pos_", "Pos-")

- Split the Var2 column on the underscore character to create 3 new columns:

("split_column", "Var2", "_", ['WellID','CpdID','PlateID'])

- Pivot the table, populating new features with the values in the ‘Var1’ column, and their values from the ‘value’ column. Further reading on pivoting can help in grasping the idea behind this transformation. See <https://pandas.pydata.org/docs/user_guide/reshaping.html>

("pivot", "Var1", 'value')

- We may optionally undo the renaming operation now that we have split the filed, and replace ‘Pos-’ with ‘Pos_’ in the ‘CpdID’ column:

("replace_str", "CpdID", "Pos-", "Pos_")

Putting it all together produces the following Phenonaut code, containing metadata, with nested transform keys:

phe = Phenonaut(

        "DRUG-seq_input_file.csv",

        metadata={

            "transforms": [

                ("replace_str", "Var2", "Pos_", "Pos"),

                ("split_column", "Var2", "_", ['WellID','CpdID','PlateID']),

                ("pivot", "Var1", 'value'),

                ("replace_str", "CpdID", "Pos-", "Pos_"),

            ],

            'features_prefix':'ENSG'

        },

    )

## Packaged datasets

Phenonaut contains a selection of public datasets, which can be accessed using classes inheriting from the PackagedDataset class. Users and developers are encouraged to write their own packaged dataset loaders using the Phenonaut framework and either submit pull requests for inclusion into Phenonaut or contact the authors.

The initial v1.0.0 release of Phenonaut contains the following packaged datasets:

- The Cancer Genome Atlas (Weinstein 2013) prepared using methods described by Lee (Lee 2021). Named “TCGA”.
- Connectivity Map (Lamb 2006). Named “CMAP”.
- The Iris dataset (Yann). Named “Iris”.
- The Breast cancer Wisconsin (diagnostic) dataset (Wisconsin). Named “BreastCancer”.

Please see Phenonaut API documentation for a full definition and argument usage of PackagedDataset derived classes.

Packaged datasets can be loaded as follows:

from phenonaut.packaged_datasets import BreastCancer

bc_packaged_dataset=BreastCancer("/tmp/")

Note the supply of a path (“/tmp/”) to the constructor of the PackagedDataset derived class. PackagedDatasets do not rely on the existence of the packaged dataset being available on the filesystem and will retrieve the dataset before writing it to the given data directory or in the case of the example above, linux’s temporary directory /tmp.

As packaged datasets may contain multiple views, a dictionary like notation can be used to access the views. Calling .keys() returns available Dataset names, and iterating on the PackagedDataset derived class returns keys, like a dictionary.

If a PackagedDataset derived class is passed to a Phenonaut object constructor, then a Phenonaut object is constructed containing all Datasets within the PackagedDataset derived class.

from phenonaut import Phenonaut

from phenonaut.packaged_datasets import BreastCancer

phe=Phenonaut(BreastCancer("/tmp/"))

print(phe.keys())

Outputs:

['Breast_Cancer']

We may then perform a visualisation on the data, by performing the following steps:

1. Remove highly correlated features using the variance inflation factor (VIF) technique built into Phenonaut.
2. Perform UMAP dimensionality reduction.
3. Produce scatter plot.

Removing highly correlated features can be achieved using the inbuilt VIF filter

from phenonaut.transforms.preparative import VIF

VIF().filter(phe[0])

With no arguments passed to filter apart from the Dataset to be filtered, a VIF cutoff of 5 is used. This reduces the number of features to from 31 to 13:

print(phe[0].features)

Outputs:

['worst area', 'mean texture', 'mean symmetry', 'texture error', 'symmetry error', 'worst symmetry', 'concavity error', 'mean smoothness', 'perimeter error', 'smoothness error', 'concave points error', 'mean fractal dimension', 'fractal dimension error']

We then apply the UMAP dimensionality reduction technique to the Dataset, using no arguments apart from the Dataset to be operated on – producing a 2D UMAP embedding:

from phenonaut.transforms import UMAP

UMAP()(phe[0])

Finally, we plot the output. To achieve this, we exploit a special property of datasets which may have a perturbation_column value set. This refers to a non-feature column which can be used to distinguish between samples for plotting and grouping purposes. First, however, we change the values in the target column from 0 and 1 to benign and malignant respectively, this enables proper labelling to be automatically applied to plots. Note that for brevity, we pass the whole Phenonaut object to scatter. This is a shortcut for directing the scatter object to use the last added dataset within a Phenonaut object for visualisation.

from phenonaut.output import Scatter

phe[0].df["target"]= phe[0].df["target"].map({0:"benign", 1:"malignant"})

phe[0].perturbation_column="target"

scatter=Scatter(title="UMAP of breast cancer dataset, VIF filtered")

scatter.add(phe)

Which produces the plot in Figure S3 showing separation between benign and malignant tumour samples.


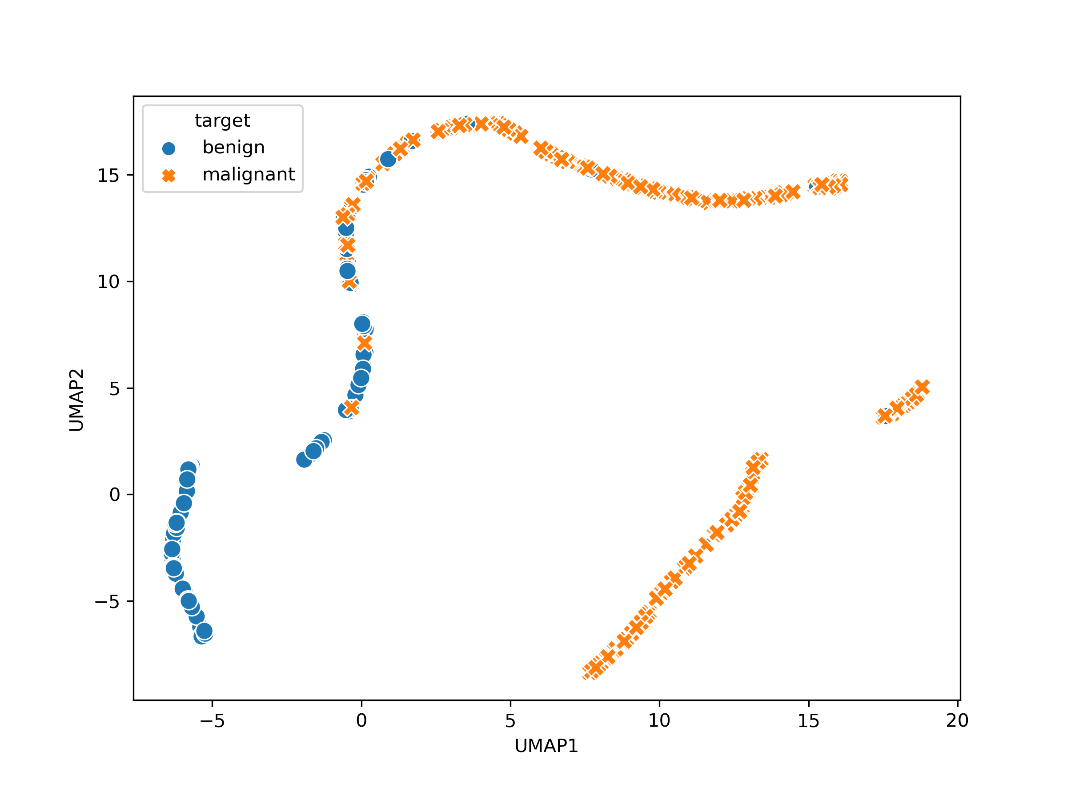


Figure S3 – Output from the scatter visualisation on the breast cancer dataset after VIF filtering and applying UMAP dimensionality reduction using default parameters and two dimensions, showing an apparent separation within feature space of benign vs malignant tumours.

# SI Example 1 – Prediction of TCGA 1 year donor survival rate.

Figure 1 in the application note manuscript contains a heatmap demonstrating performance of predictors over all possible views of The Cancer Genome Atlas (TCGA) (Weinstein 2013). The public TCGA dataset contains methylation, miRNA, mRNA and RPPA profiles and metadata for tumor samples spanning a range of clinical tumor types. A variety of metadata is also present such as the age of the donor, smoking status and time until patient death (if applicable) from when the sample was taken. The TCGA packaged dataset as present in Phenonaut uses the same preparative steps as taken by Lee (Lee 2021), whereby the dataset was downloaded, rows with missing data removed and then principal component dimensionality reduction used to reduce the feature space within each view to 100 features. In addition to those preparative steps carried out by Lee, an additional metadata column is derived from the “days_to_death” column, indicating if the tumor donor survives 1 year after the sample was taken and named ‘survives_1_year’.

The code used to direct Phenonaut in the production of the performance heatmap is available within the GitHub repository with the name “example1_TCGA.py”. This was executed in a Python 3.9.5 environment using Phenonaut version 1.0.0, Numpy version 1.20.3, Pandas version 1.4.0, scikit-learn version 0.24.2, and PyTorch version 1.10.2. Whilst the full source of example1_TCGA.py is considerably longer and more verbose than the code used to exemplify the task in Figure 1, additional checks on command line parameters and use of the fire interface module are not required. The minimal Python code listing given in Figure 1, along with the workflow YAML file are both sufficient to produce the reported output.

Within Phenonaut, the predict submodule contains a profile function with purpose of applying all suitable predictors (classifiers, regressors, or multiregressors) to all possible view combinations for a given prediction target. The profile function performs the following:

1. The data within the prediction target is examined and the prediction type determined from classification, regression, and multiregression/view prediction (prediction of 1 omics view from another). In the case of Example 1, on TCGA, with the prediction target of “survives_1_year”, the data types within the metadata are examined and only two values found 0 (no) or 1 (yes). Classification is chosen.
2. With no classifiers explicitly given as arguments to the profile function, Phenonaut selects all default classifiers. User supplied classifiers and predictors may be passed, including PyTorch neural networks and similar objects implementing fit and predict methods. See API documentation for further information on including user defined and packaged predictors.
3. With no views explicitly listed, all possible view combinations are selected. For TCGA, the four omics views allow 15 view combinations (4x singular, 6x double, 4x triple and 1x quad).
4. For each unique view combination and predictor, perform the following:
   1. Merge views and remove samples which do not have features across currently needed views.
   2. Shuffle the samples.
   3. Withhold 20% of the data as a ‘test’ set, to be tested against the trained and hyperparameter optimised predictor.
   4. Split the data using 5-fold cross validation into train and validation sets.
   5. For each fold, perform Optuna hyperparameter optimisation for the given predictor using the train sets, using hyperparameters described by the default predictors for classification, regression and multiregression (see API documentation for further information).
   6. Optimized models are then evaluated with the held-out test set and standard deviations calculated from validation sets.
5. Writing output from the profiling process consisting of performance heatmaps highlighting best view/predictor combinations in bold (Figure S4), boxplots for each view combination (Figure S5) and a PPTX presentation file allowing easy sharing of data, along with machine readable CSV and JSON results.


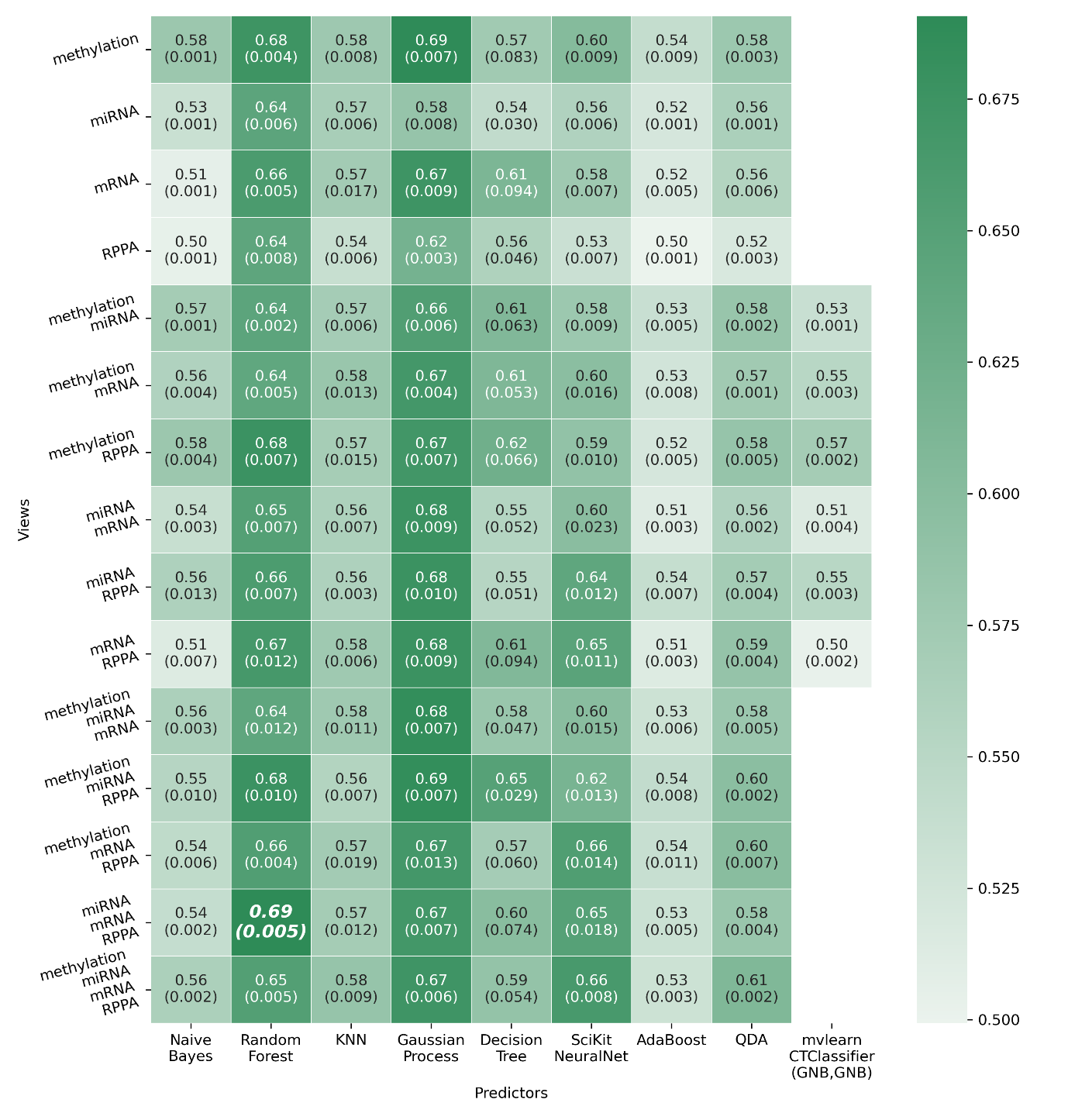


Figure S4 –Predictor-view performance heatmap showing AUROC scores, generated by calling the predict.profile function on the TCGA packaged dataset, predicting 1 year survival rates of tumor donors. Train, validation, test splitting is used, along with the Optuna hyperparameter optimisation library to optimise all given predictors, across all possible view combinations. The best scoring predictor on the best combination of views is shown in bold (miRNA, mRNA and RPPA) using a random forest predictor.


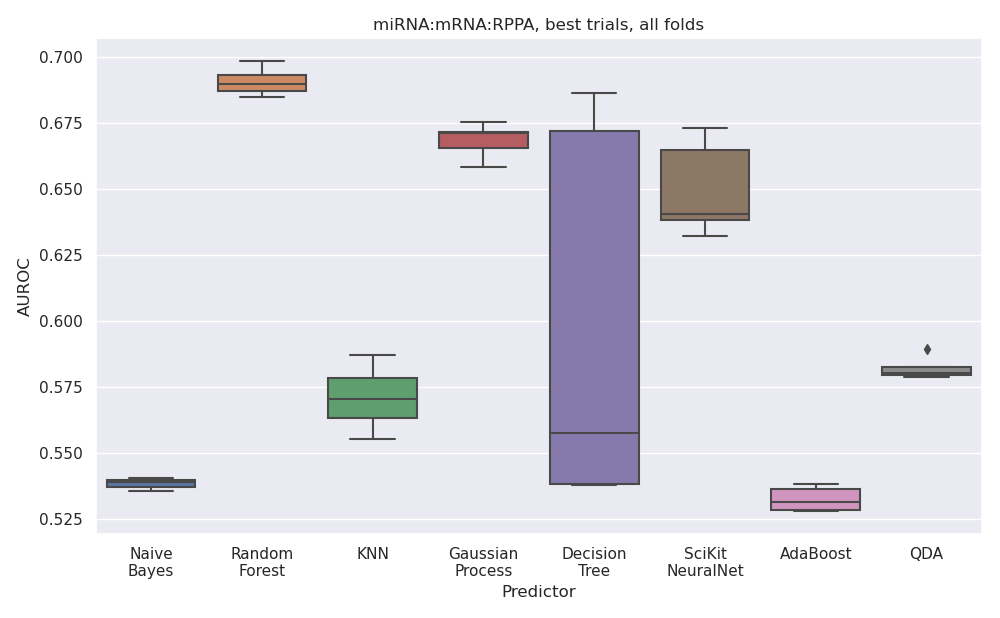


Figure S5 – Boxplot performance heatmap of all validation folds across the best view; miRNA, mRNA and RPPA, as determined by test set.

In addition to the “best view” performance boxplot shown in Figure S4, boxplots across all views are output as PNGS, and included into a summative PPTX presentation file along with the overall performance heatmap shown in Figure S5, and machine readable performance measures in the CSV and JSON file formats.

# CMAP - Exploration of the Connectivity Map

The purpose of example 2 in the lower right portion of Figure 1 in the application note manuscript was to exemplify use of the CMAP packaged dataset, along with user supplied and built-in phenotypic metrics. An interactive Jupyter notebook (Jupyter core version 4.9.1) was used to carry out this analysis with version Python 3.9.5, Phenonaut version 1.0.0, Numpy version 1.20.3, Pandas version 1.4.0, scikit-learn version 0.24.2, and PyTorch version 1.10.2 installed. The notebook can be found in the Phenonaut source repository named “example2_CMAP_metric_groups.ipynb”. The notebook was used to evaluate the built in (Euclidean and Manhattan distances) and user defined phenotypic metrics (TCCS[6] and scalar projection[7]). In this notebook, the CMAP packaged dataset loader is used to obtain a copy of the public CMAP dataset, which is then filtered, keeping only CRISPR perturbation repeats on the A549 cell line transfected with the pLX311-Cas9 vector, from here referred to using the internal CMAP nomenclature of the ‘A549.311’ cell line. The phenotypic metrics are then evaluated using Area Under the Receiver Operator Characteristic Curve (AUROC) metric, indicating their ability to group together repeats. Boxplots of the AUROC performance across repeats are then generated to give an overview of metric performance. The jupyter file “example2_CMAP_metric_groups.ipynb” available in the “Supplemental Data from Object Store” section gives the code listing required to explore and generate the boxplot shown within Figure 1 of the manuscript.

A walkthrough of the notebook follows:

CRISPR (xpr) repeats present in the CMAP dataset offer the possibility to evaluate phenotypic metrics applied to different representations of the level 5 L1000 profiles within the CMAP dataset. Here, we evaluate metric-feature space pairs and their ability to correctly rank xpr repeats as highly similar to each other. Metric and feature space pairs are scored through evaluation of the area under receiver operating characteristic (AUROC), with 0.5 representative of random guessing, 1, perfect performance, and 0 representative of perfectly incorrect ranking being applied to the crispr repeats.

We now use the CMAP package dataset loader to extract the A549.311 cell line and perform the evaluation of the following phenotypic metrics:

- Euclidean distance
- Manhattan/cityblock distance
- Cosine similarity
- Scalar projection

We evaluate these on:

- Full feature space
- Standard Scalar feature space
- PCA feature space
- t-SNE feature space
- UMAP feature space.

t-SNE and UMAP are unsuitable for the application of the cosine similarity and scalar projection, being angular metrics and are not applied.

First, use the Phenonaut CMAP dataloader to load the entire CMAP dataset. Process it, extracting the A549.311 cell line where treatment is ‘xpr peturbation’ or ‘ctl_untrt’.

Once processed, save it in a pickle so that it can more quickly be loaded in the future. As a "standard scaler" feature space is required for input to PCA, UMAP and t-SNE, we generate standard scalar dataset. Next, we define the similarity metrics and scoring function.

In the code below, BASE_PROJECT_DIR took the location of a convenient output directory on the local filesystem.

import numpy as np

import pandas as pd

import phenonaut, phenonaut.transforms

from pathlib import Path

BASE_PROJECT_DIR=Path("projects/cmap")

if not BASE_PROJECT_DIR.exists():

    BASE_PROJECT_DIR.mkdir(parents=True)

phe_object_path=BASE_PROJECT_DIR/"phe_ob_cmap_trt_xpr_A549.311.pickle.gz"

if not phe_object_path.exists():

    cmap_data=phenonaut.packaged_datasets.CMAP("/local_scratch/data/phenonaut_datasets/cmap")

    phe=phenonaut.Phenonaut(cmap_data['ds'])

    phe.new_dataset_from_query("xpr", "cell_id=='A549.311' and (pert_type=='trt_xpr' or pert_type=='ctl_untrt')", "cmap")

    phe['xpr'].perturbation_column="pert_iname"

    phe.save(phe_object_path)

phe=phenonaut.Phenonaut.load(phe_object_path)

phe.clone_dataset("xpr", "xpr_scaled",overwrite_existing=True)

phenonaut.transforms.StandardScaler()(phe['xpr_scaled'])

from scipy.spatial.distance import cosine as cosine_distance

from dataclasses import dataclass

from typing import Union, Callable

@dataclass

class PhenotypicMetric:

    name: str

    func: Union[str, Callable]

    lower_is_better: bool = True

    is_angular: bool = False

metrics = [

    PhenotypicMetric("random", lambda x, y: np.random.uniform(0, 1)),

    PhenotypicMetric("euclidean", "euclidean"),

    PhenotypicMetric("Manhattan", "cityblock"),

    PhenotypicMetric("cosine similarity", lambda x, y: 1 - cosine_distance(x, y), lower_is_better = False, is_angular=True),

    PhenotypicMetric("scalar projection", phenonaut.metrics._scalar_projection_scaled, lower_is_better = False, is_angular=True),

]

metrics_dictionary = {m.name: m for m in metrics}

from sklearn.metrics import roc_auc_score

def auroc_scores_for_perturbation_repeats(ds:phenonaut.data.Dataset, metric:PhenotypicMetric, average_repeats:bool=True)->tuple:

    pert_scores=[]

    df_dict = {"score": [], "perturbation": []}

    dist_mat = ds.distance_df(

        ds,

        metric=metric.func,

        lower_is_better=metric.lower_is_better,

    ).values

    for pert_name in ds.df[ds.perturbation_column].unique():

        pert_indexes = [ds.df.index.get_loc(idx) for idx in ds.df.query(f"pert_iname == '{pert_name}'").index]

        repeat_scores = []

        if metric.lower_is_better:

            for pert_index in pert_indexes:

                predictions = np.zeros(dist_mat.shape[1], dtype=int)

                predictions[pert_indexes] = 1

                prediction_score = -dist_mat[pert_index, :]

                rocscore = roc_auc_score(predictions, prediction_score)

                repeat_scores.append(rocscore)

        else:

            for pert_index in pert_indexes:

                predictions = np.zeros(dist_mat.shape[1], dtype=int)

                predictions[pert_indexes] = 1

                prediction_score = dist_mat[pert_index, :]

                rocscore = roc_auc_score(predictions, prediction_score)

                repeat_scores.append(rocscore)

        if average_repeats:

            df_dict['score'].append(np.mean(repeat_scores))

            df_dict['perturbation'].append(pert_name)

        else:

            df_dict['score'].extend(repeat_scores)

            df_dict['perturbation'].extend([pert_name]*len(repeat_scores))

        pert_scores.append(np.mean(repeat_scores))

    return pert_scores, pd.DataFrame(df_dict)

Next, we generate PCA, UMAP, t-SNE using the standard scalar feature space. ndims = 2 at this stage so we can obtain a unoptimized baseline.

from phenonaut.transforms.dimensionality_reduction import PCA,TSNE,UMAP

NUM_DIMENSIONS=2

phe.clone_dataset("xpr", "xpr_scaled",overwrite_existing=True)

phenonaut.transforms.StandardScaler()(phe['xpr_scaled'])

phe.clone_dataset("xpr_scaled", "xpr_scaled_pca",overwrite_existing=True)

phe.clone_dataset("xpr_scaled", "xpr_scaled_umap",overwrite_existing=True)

phe.clone_dataset("xpr_scaled", "xpr_scaled_tsne",overwrite_existing=True)

pca=PCA()

# Centering on the untreated samples is a good way to place untreated at origin of new reduced feature space.

# Set to None to not perform the recentring.

center_on_perturbation="UnTrt"

pca(phe["xpr_scaled_pca"], ndims=NUM_DIMENSIONS,center_on_perturbation_id=center_on_perturbation, explain_variance_in_features=True)

TSNE()(phe["xpr_scaled_tsne"],ndims=NUM_DIMENSIONS,center_on_perturbation_id=center_on_perturbation)

UMAP()(phe["xpr_scaled_umap"],ndims=NUM_DIMENSIONS, center_on_perturbation_id=center_on_perturbation)

In order to perform a sanity check, the xpr repeats were visualised in PCA, tSNE and UMAP space.

ds=phe['xpr_scaled_pca']

scatter=phenonaut.output.Scatter(title="PCA scatter - A549.311 Xpr")

scatter.add(ds, marker_size=90,perturbations=['BRAF', 'EGFR', 'ERBB3', 'JUN', 'KRAS', 'SMAD4'])

scatter.add(ds, marker_size=90,perturbations=['UnTrt'], markers="X")

scatter.add(ds,perturbations=['MAST4'], markers="P", marker_size=300)

scatter._decorate_figure()

scatter.save_figure(BASE_PROJECT_DIR/"pca_scatter.png", dpi=200)

scatter=phenonaut.output.Scatter(title="UMAP scatter - A549.311 Xpr")

ds=phe['xpr_scaled_umap']

scatter.add(ds, marker_size=90,perturbations=['BRAF', 'EGFR', 'ERBB3', 'JUN', 'KRAS', 'SMAD4'])

scatter.add(ds, marker_size=90,perturbations=['UnTrt'], markers="X")

scatter.add(ds,perturbations=['MAST4'], markers="P", marker_size=200)

scatter._decorate_figure()

scatter.save_figure(BASE_PROJECT_DIR/"umap_scatter.png", dpi=200)

scatter=phenonaut.output.Scatter(title="t-SNE scatter - A549.311 Xpr")

ds=phe['xpr_scaled_tsne']

scatter.add(ds, marker_size=90,perturbations=['BRAF', 'EGFR', 'ERBB3', 'JUN', 'KRAS', 'SMAD4'])

scatter.add(ds, marker_size=90,perturbations=['UnTrt'], markers="X")

scatter.add(ds,perturbations=['MAST4'], markers="P", marker_size=200)

scatter._decorate_figure()

scatter.save_figure(BASE_PROJECT_DIR/"tsne_scatter.png", dpi=200)

Producing Figure S6, Figure S7, and Figure S8.


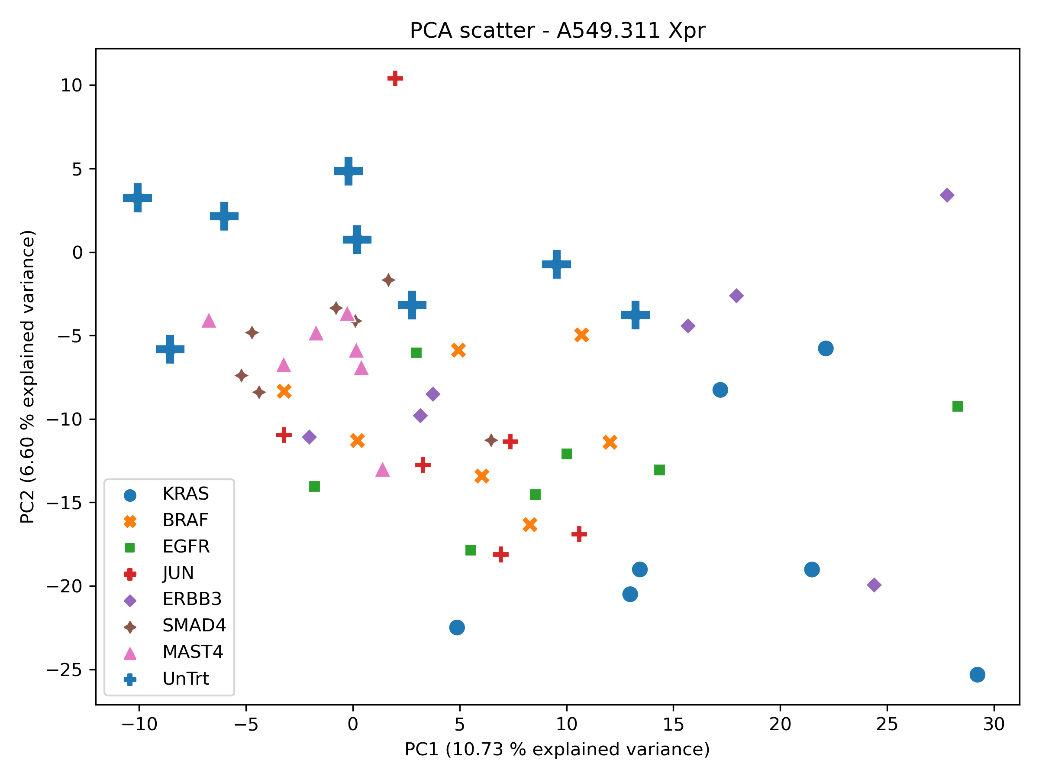


Figure S6 - PCA scatter of CMAP A549.311 cell line CRISPR repeats. Large blue ‘+’ are untreated controls.


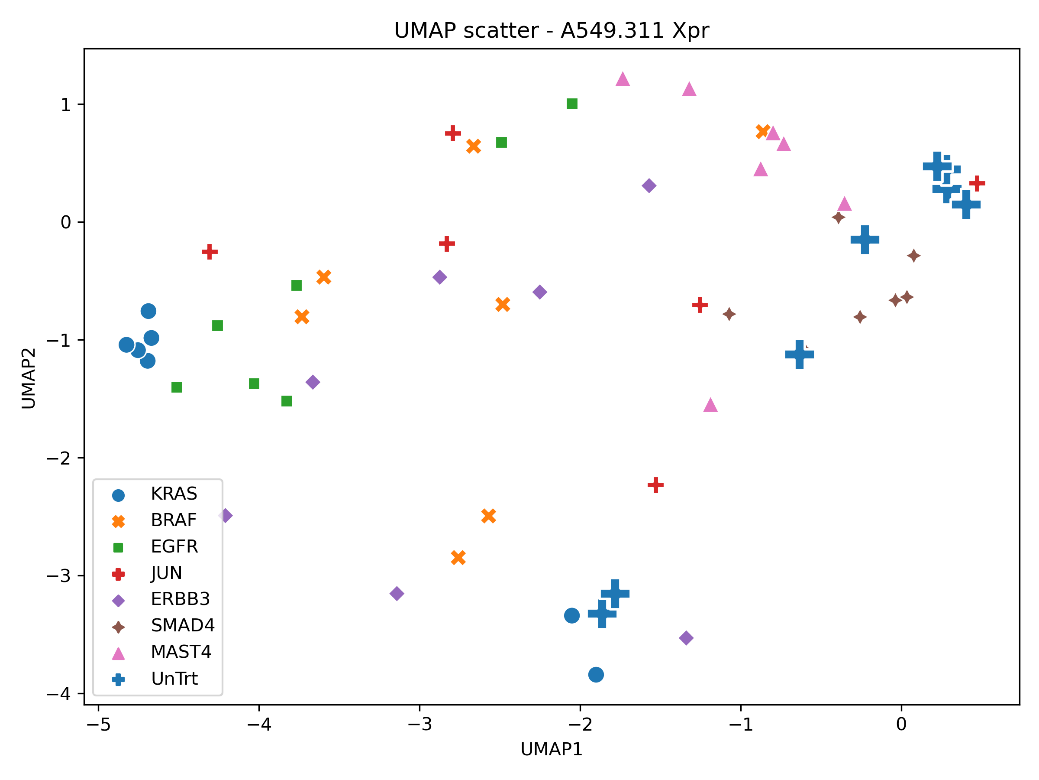


Figure S7 - UAMP scatter of CMAP A549.311 cell line CRISPR repeats. Large blue ‘+’ are untreated controls.


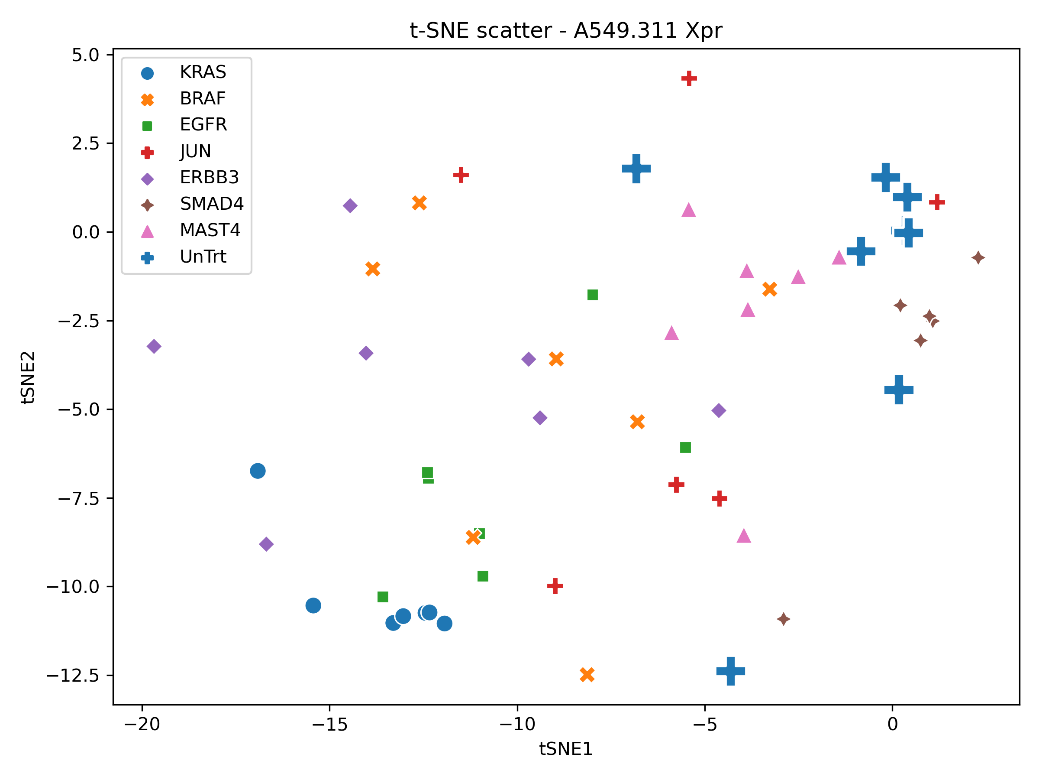


Figure S8 – t-SNE scatter of CMAP A549.311 cell line CRISPR repeats. Large blue ‘+’ are untreated controls.

With local structure present within the UMAP and tSNE scatters for a selection of xpr perturbations which roughly group together, the dataset was deemed appropriate for phenotypic metric performance evaluation.

The following code is used to generate AUROC scores across xpr repeats for appropriate feature space-metric pairs:

if (BASE_PROJECT_DIR/"metric_scores.csv").exists():

    raise FileExistsError(f"{BASE_PROJECT_DIR}/metric_scores.csv exists")

perturbation_scores_df=pd.DataFrame()

for features_name, ds_name in zip(

    ("Full", "Std", "PCA", "UMAP", "t-SNE"),

    ("xpr", "xpr_scaled", "xpr_scaled_pca", "xpr_scaled_umap", "xpr_scaled_tsne"),

):

    print(f"Working on {features_name}")

    for metric in metrics:

        if metric.is_angular and features_name in ['UMAP','t-SNE']:

            continue

        print(f"{metric.name=}")

        scores, feature_metric_df=auroc_scores_for_perturbation_repeats(phe[ds_name],metric)

        feature_metric_df["metric_name"]=metric.name

        feature_metric_df["features"]=features_name

        perturbation_scores_df=pd.concat([perturbation_scores_df, feature_metric_df]).reset_index(drop=True)

perturbation_scores_df.to_csv(BASE_PROJECT_DIR/"metric_scores.csv")

A boxplot was then generated to summarise the results of the above metric evaluation:

import matplotlib.pyplot as plt

import seaborn as sns

metric_scores_df=pd.read_csv(BASE_PROJECT_DIR/"metric_scores.csv")

fig, ax = plt.subplots(1, facecolor='w')

# the size of A4 paper

fig.set_size_inches(12.0, 8)

sns.boxplot(

    data=metric_scores_df,

    y="score",

    x="metric_name",

    hue="features",

    ax=ax,

    showmeans=True,

    meanprops={"marker": "o", "markerfacecolor": "white", "markeredgecolor": "black", "markersize": "10"},

)

ax.axhline(0.5, color="black", linestyle="--")

ax.set(ylim=(0.2,1), xlabel='Metric', ylabel='AUROC', title="AUROC scores of average xpr repeat reproducibility across phenotypic metrics and feature spaces")

plt.tight_layout()

fig.savefig(BASE_PROJECT_DIR/"boxplot.png", dpi=300)

Resulting in production of Figure S9. Whilst distance measurements in UMAP and TSNE space should be avoided due to their dependence on hyperparameters, this is performed for demonstration purposes.


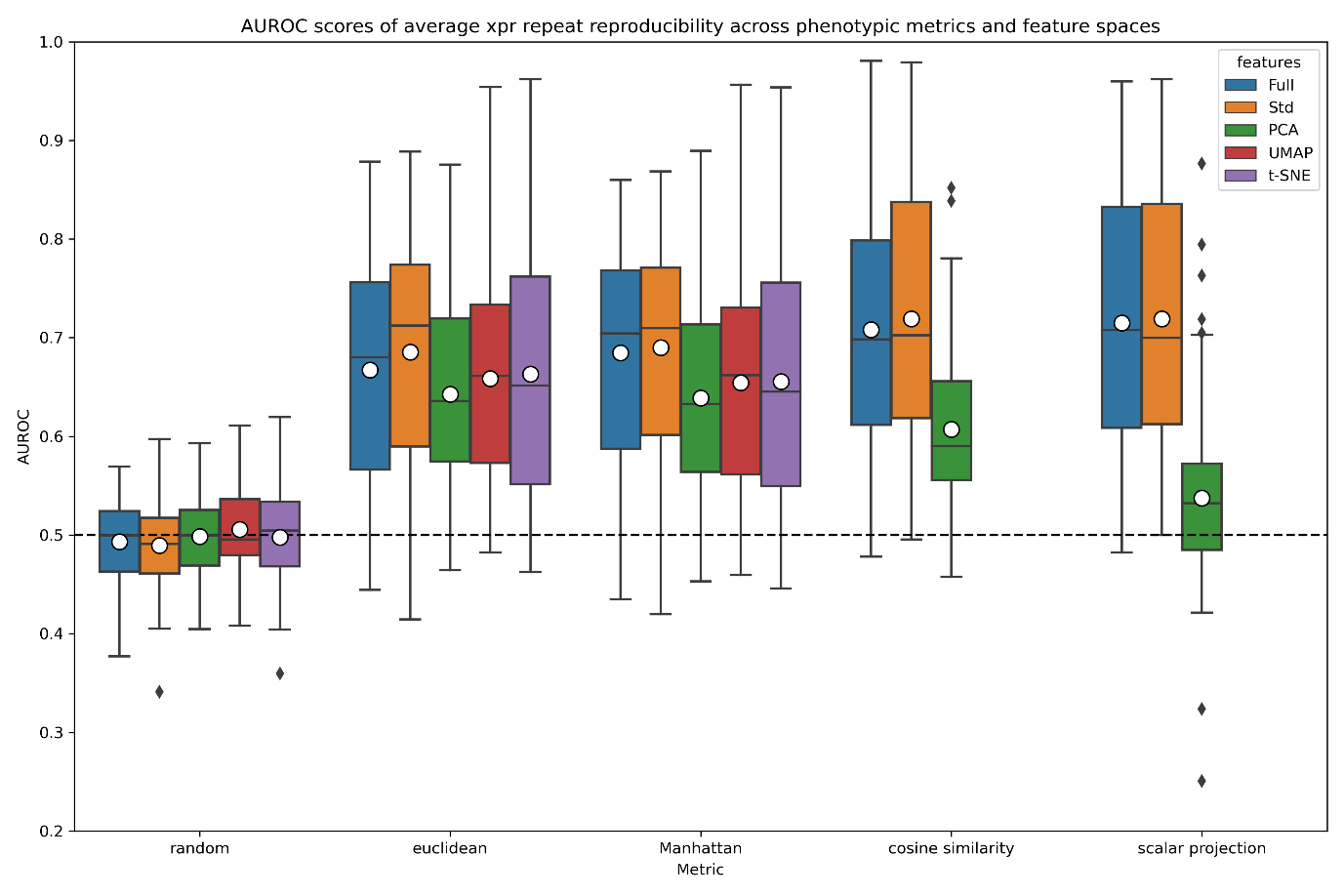


Figure S9 – Boxplot of AUROC scores across averaged xpr repeats of the A549.311 cell line within CMAP. For each dimensionality reduction technique (PCA, UMAP and tSNE), 2 dimensions were requested, and all other options kept to default settings.

Figure S9 gives us an insight into metric-feature space performance, but statistically testing all distributions against the others will allow us to recommend a metric-feature space pair. For this, we use the Mann-Whitney U rank test in a 1-tailed mode, testing the alternative hypothesis that a distribution is stochastically greater than the other. Once calculated, two representative heatmaps can be generated. The code below produces a matrix as shown in Figure S10, and summarised in Figure S11.

metric_scores_df=pd.read_csv(BASE_PROJECT_DIR/"metric_scores.csv")

@dataclass

class FeatureSpaceAndMetric():

    featurespace:str

    metric:PhenotypicMetric

from scipy.stats import mannwhitneyu

featurespeace_metric_combinations=[FeatureSpaceAndMetric(feature_space, metric) for feature_space in ("Full", "Std", "PCA", "UMAP", "t-SNE") for metric in [m for m in metrics if m.name !="random"] if not (metric.is_angular and feature_space in ['UMAP', 't-SNE'])]

pvals=np.full((len(featurespeace_metric_combinations), len(featurespeace_metric_combinations)),np.nan)

for i1, fsm1 in enumerate(featurespeace_metric_combinations):

    for i2, fsm2 in enumerate(featurespeace_metric_combinations):

        vals1=metric_scores_df.query(f"features=='{fsm1.featurespace}' and metric_name=='{fsm1.metric.name}'")['score'].values

        vals2=metric_scores_df.query(f"features=='{fsm2.featurespace}' and metric_name=='{fsm2.metric.name}'")['score'].values

        pvals[i1, i2]=mannwhitneyu(vals1, vals2,alternative="greater").pvalue

mwu_df=pd.DataFrame(data=pvals, columns=[f"{fsm.featurespace} {fsm.metric.name}" for fsm in featurespeace_metric_combinations], index=[f"{fsm.featurespace} {fsm.metric.name}" for fsm in featurespeace_metric_combinations])

from phenonaut.output import write_heatmap_from_df

write_heatmap_from_df(mwu_df,"1-tailed (greater) Mann-Whitney U tests. P values for query metric-features pair AUROC scores being the same or less than candidate metric-features pair AUROC scores", BASE_PROJECT_DIR/"mwu-heatmap.png", figsize=(20,16), lower_is_better=True, highlight_best=False, put_cbar_on_left=True, axis_labels=("Candidate metric-features pair A", "Query metric-features pair"))

better_than_count=np.sum(pvals[:]<0.05,axis=1)

better_than_count=np.insert(better_than_count,-2, [0,0])

better_than_count=np.insert(better_than_count,better_than_count.shape[0], [0,0]).reshape(5,4)

better_than_df=pd.DataFrame(data=better_than_count, index=("Full", "Std", "PCA", "UMAP", "t-SNE"), columns=[m.name for m in metrics if m.name !="random"])

custom_annotation=better_than_df.copy()

custom_annotation.loc[['UMAP','t-SNE'], ['scalar projection', 'cosine similarity']]="NA"

write_heatmap_from_df(better_than_df,"Num significant at 0.05 level", BASE_PROJECT_DIR/"mwu-betterthan-heatmap.png", figsize=(5,4), lower_is_better=False, annotation_format_string_value="g", annot=custom_annotation.transpose().values, sns_colour_palette="Blues")


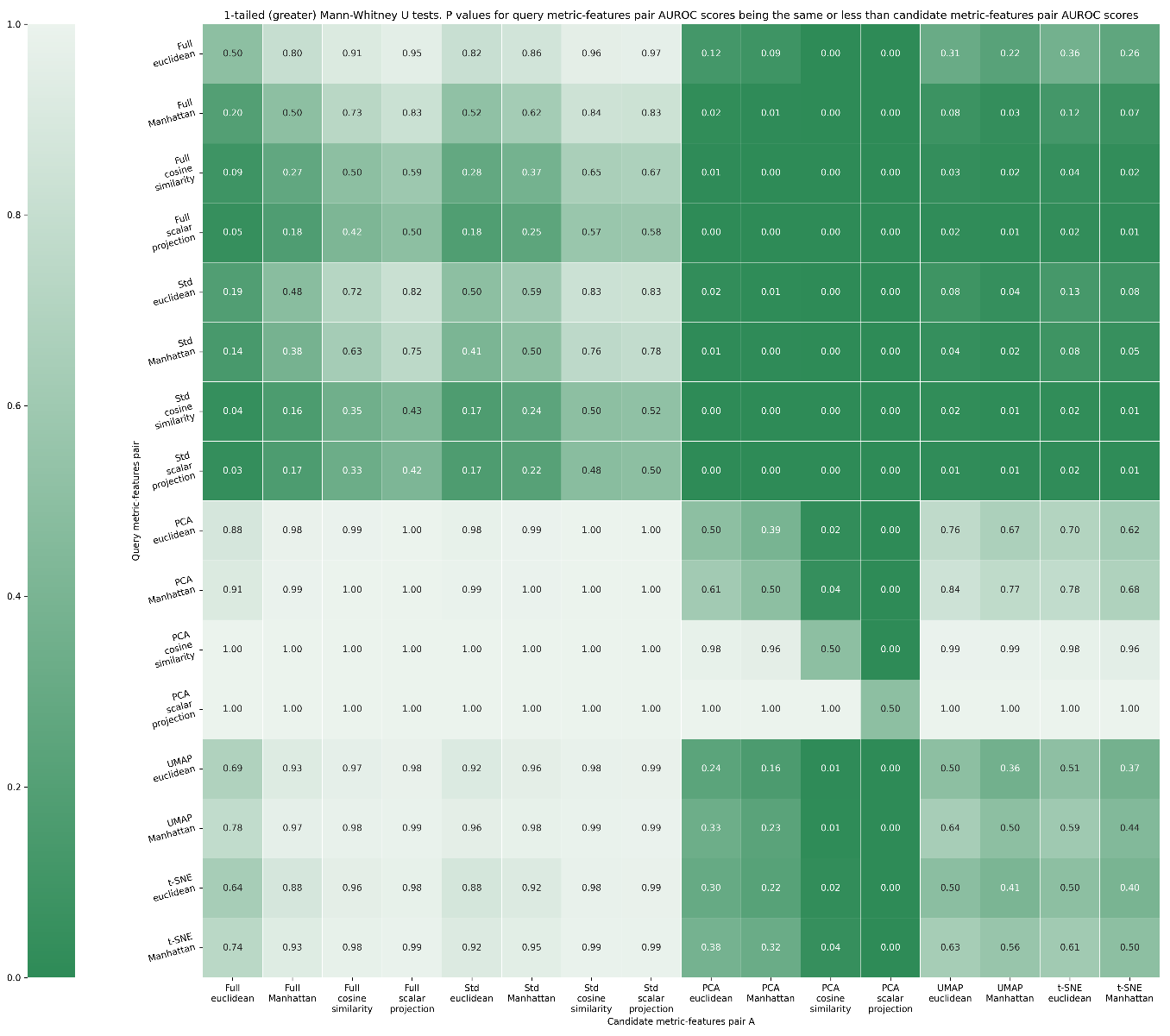


Figure S10 – 1-tailed Mann Whitney-U (greater than) test, evaluating if the query metric-feature space combination is better than the candidate-feature space combination. Values denote p-values that the metric-feature space combination in a given row is performed better than the metric-feature space column given in the column by chance along. If this value is <0.05, we deem there to be a less than 5 % chance that the metric-feature space combination outperformed the other by chance.


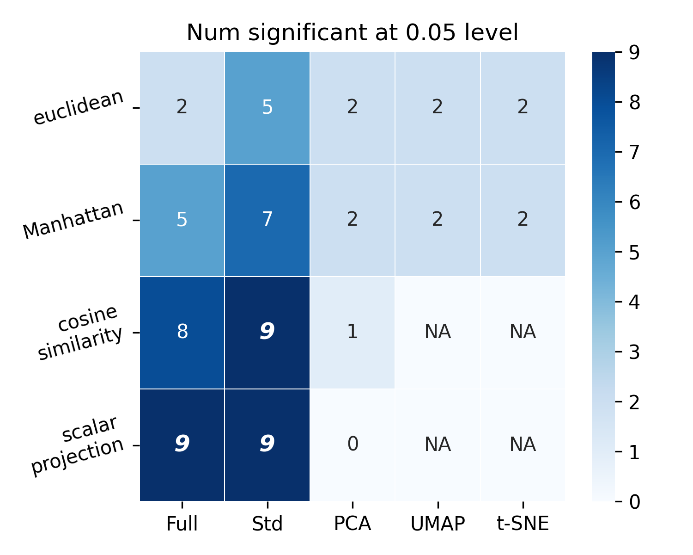


Figure S11 - Counts of the number of features-space metric pairs that were significantly better performing than other metric-feature space pairs. Suggests that scalar projection applied to full and standard scalar feature space outperforms the same number of metric-feature space pairs as cosine similarity applied to standard scalar feature space.

Whilst Figure S9 shows that scalar projection applied to full and standard scalar feature space outperforms the same number of metric-feature space pairs as cosine similarity applied to standard scalar feature space, there is no significant (at the 0.05 level) performance difference between any of those top metric-feature space combinations, as evidenced in Figure S10.

Additionally, the PCA, UMAP and t-SNE feature spaces are in 2 dimensions only. This can be optimised using a simple scanning approach to reach optimum AUROC scores for each metric-feature space pair.

from dataclasses import dataclass

from phenonaut.transforms.dimensionality_reduction import PCA,TSNE,UMAP

from typing import Union, Callable

import matplotlib.pyplot as plt

import seaborn as sns

from enum import Enum, auto

ndims_scan_csv_file=BASE_PROJECT_DIR/"res_ndims_scan.csv"

if not ndims_scan_csv_file.exists():

    def eval_all_metrics(sds, num_dimensions):

        print(f"{num_dimensions=}")

        center_on_perturbation = "UnTrt"

        pca_ds=sds.copy()

        PCA()(pca_ds, ndims=num_dimensions, center_on_perturbation_id=center_on_perturbation)

        tsne_ds=sds.copy()

        TSNE()(tsne_ds, ndims=num_dimensions, center_on_perturbation_id=center_on_perturbation)

        umap_ds=sds.copy()

        UMAP()(umap_ds, ndims=num_dimensions, center_on_perturbation_id=center_on_perturbation)

        results=np.empty((len([m for m in metrics if m.name != "random"]), 3))

        for i, metric in enumerate([m for m in metrics if m.name != "random"]):

            for j, (ds, angular_is_ok) in enumerate(((pca_ds, True), (tsne_ds, False), (umap_ds, False))):

                if not metric.is_angular or (metric.is_angular and angular_is_ok):

                    auroc_scores, _ = auroc_scores_for_perturbation_repeats(ds,metric)

                    results[i,j]=np.mean(auroc_scores)

                else:

                    results[i,j]=np.nan

        return results

    from multiprocessing import Pool

    sds = phe["xpr_scaled"].copy()

    n_dims_list = np.arange(2, min(sds.df.shape[0]-2, sds.df.shape[1]-2))

    print(f"{len(n_dims_list)=}")

    # [PCA, tSNE, UMAP], metric, dimensions

    pool = Pool()

    res = np.array(pool.starmap(eval_all_metrics, ((sds, nd) for nd in n_dims_list)))

    pool.close() # ATTENTION HERE

    df_metric_list=[]

    df_features_list=[]

    df_ndims_list=[]

    df_score_list=[]

    for i in range(res.shape[0]):

        for j in range(res.shape[1]):

            for k in range(res.shape[2]):

                print(i,j,k)

                df_ndims_list.append(i+2)

                df_metric_list.append([m for m in metrics if m.name != "random"][j].name)

                df_features_list.append(["PCA", "tSNE", "UMAP"][k])

                df_score_list.append(res[i,j,k])

    import pandas as pd

    pd.DataFrame({"metric":df_metric_list, "features":df_features_list, "ndims":df_ndims_list, "score":df_score_list}).to_csv(ndims_scan_csv_file)

# CSV file exists or has been generated above

ndims_df=pd.read_csv(ndims_scan_csv_file)

fig, ax=plt.subplots(1,3, figsize=(16,7), sharey=True)

sns.scatterplot(data=ndims_df.query('features=="PCA"'), x='ndims', y="score", style='metric', hue='metric', ax=ax[0], legend=True)

ax[0].title.set_text('PCA - ndims vs AUROC score')

sns.scatterplot(data=ndims_df.query('features=="UMAP" and metric!="cosine similarity" and metric!="scalar projection"'), x='ndims', y="score", style='metric', hue='metric', ax=ax[1], legend=True)

ax[1].title.set_text('UMAP - ndims vs AUROC score')

sns.scatterplot(data=ndims_df.query('features=="tSNE" and metric!="cosine similarity" and metric!="scalar projection"'), x='ndims', y="score", style='metric', hue='metric', ax=ax[2],legend=True)

ax[2].title.set_text('t-SNE - ndims vs AUROC score')

lines_labels = [ax.get_legend_handles_labels() for ax in fig.axes]

lines, labels = [sum(lol, []) for lol in zip(*lines_labels)]

for axis in ax: sns.move_legend(axis, "lower right")

plt.tight_layout()

fig.savefig(BASE_PROJECT_DIR/"ndims_optimisation_scatter.png", dpi=300)

This produces the scatter shown in Figure S12.


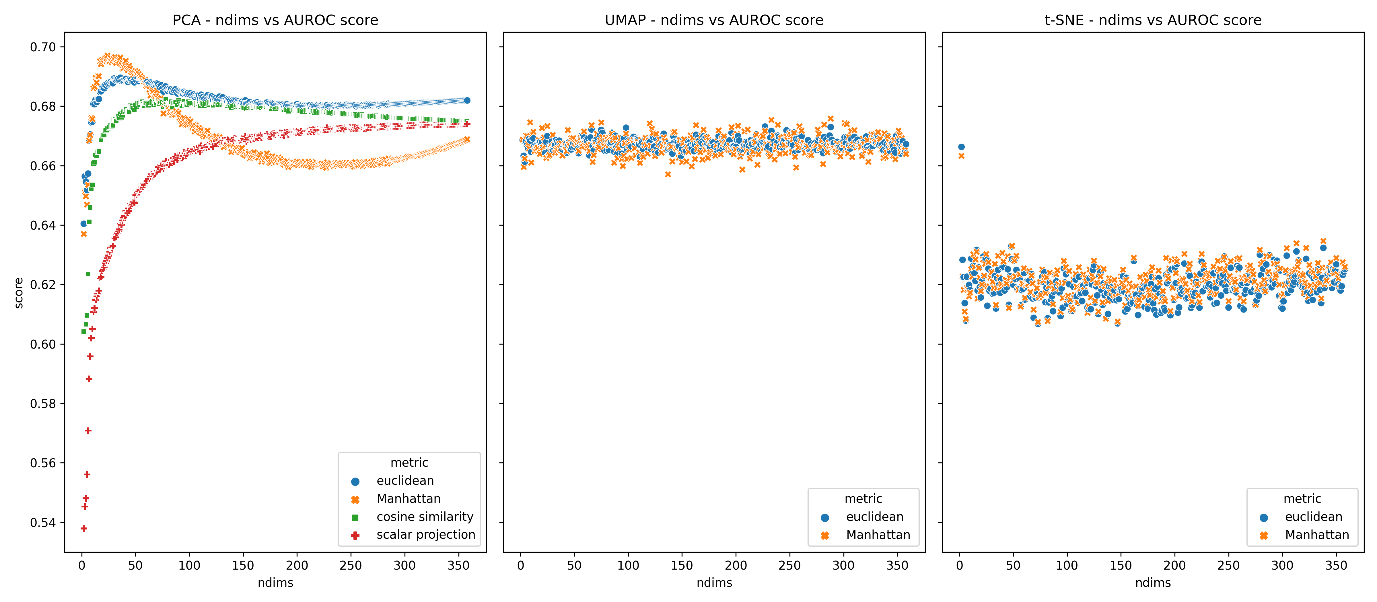


Figure S12 – AUROC scores for PCA, UMAP and t-SNE feature spaces across a range of dimensions for each appropriate metric.

The top performing ndims by virtue of giving the highest AUROC score (in absence of significance testing) for each metric-feature space are summarised in Table S4.

Table S4 – Optimal number of dimensions (maximising AUROC scores) for each metric-feature space pair (AUROC).

| Metric | PCA optimized ndims | UMAP optimized ndims | t-SNE optimized ndims |
| --- | --- | --- | --- |
| Euclidean | 36 | 227 | 2 |
| Manhattan | 24 | 288 | 2 |
| Cosine similarity | 78 | NA | NA |
| Scalar projection | 356 | NA | NA |

With the optimum number of dimensions for metric-feature space pairs determined, we may calculate AUROC scores again.

metric_scores = {"metric_name": [], "score": [], "features": []}

metrics_dict = {m.name: m for m in metrics}

perturbation_scores_ndim_opt=pd.DataFrame()

print(metrics_dict)

ds_pca_36 = phe["xpr_scaled"]).copy()

PCA()(ds_pca_36, ndims=36, center_on_perturbation_id=center_on_perturbation)

ds_pca_24 = phe["xpr_scaled"].copy()

PCA()(ds_pca_24, ndims=24, center_on_perturbation_id=center_on_perturbation)

ds_pca_78 = phe["xpr_scaled"].copy()

PCA()(ds_pca_78, ndims=78, center_on_perturbation_id=center_on_perturbation)

ds_pca_356 = phe["xpr_scaled"].copy()

PCA()(ds_pca_356, ndims=356, center_on_perturbation_id=center_on_perturbation)

ds_tsne_2 = phe["xpr_scaled"].copy()

TSNE()(ds_tsne_2, ndims=2)

ds_umap_227 = phe["xpr_scaled"].copy()

UMAP()(ds_umap_227, ndims=227)

ds_umap_288 = phe["xpr_scaled"].copy()

UMAP()(ds_umap_288, ndims=288)

for features_name, ds, metric in [

    ("Full", phe["xpr"].copy(), metrics_dict["random"]),

    ("Std", phe["xpr_scaled"].copy(), metrics_dict["random"]),

    ("PCA", ds_pca_24, metrics_dict["random"]),

    ("UMAP", ds_umap_227, metrics_dict["random"]),

    ("t-SNE", ds_tsne_2, metrics_dict["random"]),

    ("Full", phe["xpr"].copy(), metrics_dict["euclidean"]),

    ("Std", phe["xpr_scaled"].copy(), metrics_dict["euclidean"]),

    ("PCA", ds_pca_36, metrics_dict["euclidean"]),

    ("UMAP", ds_umap_227, metrics_dict["euclidean"]),

    ("t-SNE", ds_tsne_2, metrics_dict["euclidean"]),

    ("Full", phe["xpr"].copy(), metrics_dict["Manhattan"]),

    ("Std", phe["xpr_scaled"].copy(), metrics_dict["Manhattan"]),

    ("PCA", ds_pca_24, metrics_dict["Manhattan"]),

    ("UMAP", ds_umap_288, metrics_dict["Manhattan"]),

    ("t-SNE", ds_tsne_2, metrics_dict["Manhattan"]),

    ("Full", phe["xpr"].copy(), metrics_dict["cosine similarity"]),

    ("Std", phe["xpr_scaled"].copy(), metrics_dict["cosine similarity"]),

    ("PCA", ds_pca_78, metrics_dict["cosine similarity"]),

    ("Full", phe["xpr"].copy(), metrics_dict["scalar projection"]),

    ("Std", phe["xpr_scaled"].copy(), metrics_dict["scalar projection"]),

    ("PCA", ds_pca_356, metrics_dict["scalar projection"]),

]:

    print(f"Working on {features_name}")

    scores, feature_metric_df=auroc_scores_for_perturbation_repeats(ds,metric)

    feature_metric_df["metric_name"]=metric.name

    feature_metric_df["features"]=features_name

    perturbation_scores_ndim_opt=pd.concat([perturbation_scores_ndim_opt, feature_metric_df]).reset_index(drop=True)

perturbation_scores_ndim_opt.to_csv(BASE_PROJECT_DIR / "optimised_perturbation_scores_ndim_opt.csv")

We may then visualise the AUROC scores as shown in Figure S13.

import matplotlib.pyplot as plt

import seaborn as sns

data = pd.read_csv(BASE_PROJECT_DIR / "optimised_perturbation_scores_ndim_opt.csv")

# data['score']=data['score'].clip(upper=12000)

fig, ax = plt.subplots(1, facecolor='w')

# the size of A4 paper

fig.set_size_inches(12.0, 8)

sns.boxplot(

    data=data,

    y="score",

    x="metric_name",

    hue="features",

    ax=ax,

    showmeans=True,

    meanprops={"marker": "o", "markerfacecolor": "white", "markeredgecolor": "black", "markersize": "10"},

)

ax.axhline(0.5, color="black", linestyle="--")

data.to_csv(BASE_PROJECT_DIR/"scores.csv")

ax.set(ylim=(0.2,1), xlabel='Metric', ylabel='AUROC')

plt.tight_layout()

fig.savefig(BASE_PROJECT_DIR/"boxplot_opt.png", dpi=300)


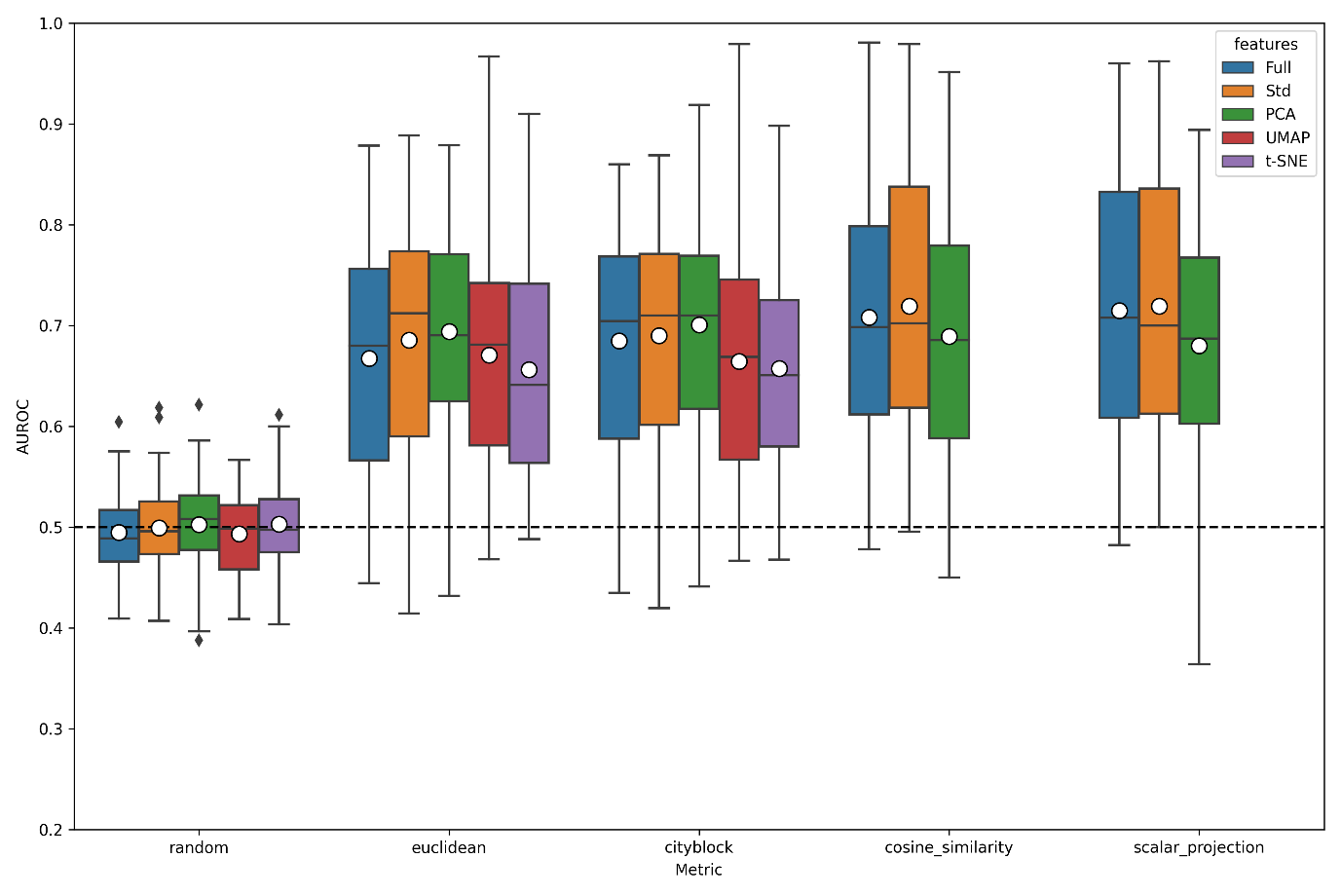


Figure S13 - Boxplot of AUROC scores across averaged xpr repeats of the A549.311 cell line within CMAP. For each dimensionality reduction technique (PCA, UMAP and tSNE), the optimum number of dimensions were used to maximise AUROC scores, indicating the theoretical maximum scores achievable using these metric-feature space pairs, other options kept to default settings.

Similarly to before, we may repeat the statistical testing to evaluate metric-feature space pair performance with each other, producing Figure S14 and Figure S15.

from scipy.stats import mannwhitneyu

optimised_perturbation_scores_df=pd.read_csv(BASE_PROJECT_DIR/"optimised_perturbation_scores.csv")

@dataclass

class FeatureSpaceAndMetric():

    featurespace:str

    metric:PhenotypicMetric

from scipy.stats import mannwhitneyu

featurespeace_metric_combinations=[FeatureSpaceAndMetric(feature_space, metric) for feature_space in ("Full", "Std", "PCA", "UMAP", "t-SNE") for metric in [m for m in metrics if m.name !="random"] if not (metric.is_angular and feature_space in ['UMAP', 't-SNE'])]

pvals=np.full((len(featurespeace_metric_combinations), len(featurespeace_metric_combinations)),np.nan)

for i1, fsm1 in enumerate(featurespeace_metric_combinations):

    for i2, fsm2 in enumerate(featurespeace_metric_combinations):

        vals1=optimised_perturbation_scores_df.query(f"features=='{fsm1.featurespace}' and metric_name=='{fsm1.metric.name}'")['score'].values

        vals2=optimised_perturbation_scores_df.query(f"features=='{fsm2.featurespace}' and metric_name=='{fsm2.metric.name}'")['score'].values

        pvals[i1, i2]=mannwhitneyu(vals1, vals2,alternative="greater").pvalue

mwu_df=pd.DataFrame(data=pvals, columns=[f"{fsm.featurespace} {fsm.metric.name}" for fsm in featurespeace_metric_combinations], index=[f"{fsm.featurespace} {fsm.metric.name}" for fsm in featurespeace_metric_combinations])

from phenonaut.output import write_heatmap_from_df

#write_heatmap_from_df(mwu_df,"1-tailed (greater) Mann-Whitney U", BASE_PROJECT_DIR/"mwu_heatmap_opt.png", figsize=(20,16), lower_is_better=True, highlight_best=False)

write_heatmap_from_df(mwu_df,"1-tailed (greater) Mann-Whitney U tests. P values for query metric-features pair AUROC scores being the same or less than candidate metric-features pair AUROC scores", BASE_PROJECT_DIR/"mwu-heatmap.png", figsize=(20,16), lower_is_better=True, highlight_best=False, put_cbar_on_left=True, axis_labels=("Candidate metric-features pair", "Query metric-features pair"), sns_colour_palette="Blues")

better_than_count=np.sum(pvals[:]<0.05,axis=1)

better_than_count=np.insert(better_than_count,-2, [0,0])

better_than_count=np.insert(better_than_count,better_than_count.shape[0], [0,0]).reshape(5,4)

better_than_df=pd.DataFrame(data=better_than_count, index=("Full", "Std", "PCA", "UMAP", "t-SNE"), columns=[m.name for m in metrics if m.name !="random"])

custom_annotation=better_than_df.copy()

custom_annotation.loc[['UMAP','t-SNE'], ['scalar projection', 'cosine similarity']]="NA"

write_heatmap_from_df(better_than_df,"Num significant at 0.05 level", BASE_PROJECT_DIR/"betterthan_heatmap_opt.png", figsize=(5,4), lower_is_better=False, annotation_format_string_value="g", annot=custom_annotation.transpose().values, sns_colour_palette="Blues")


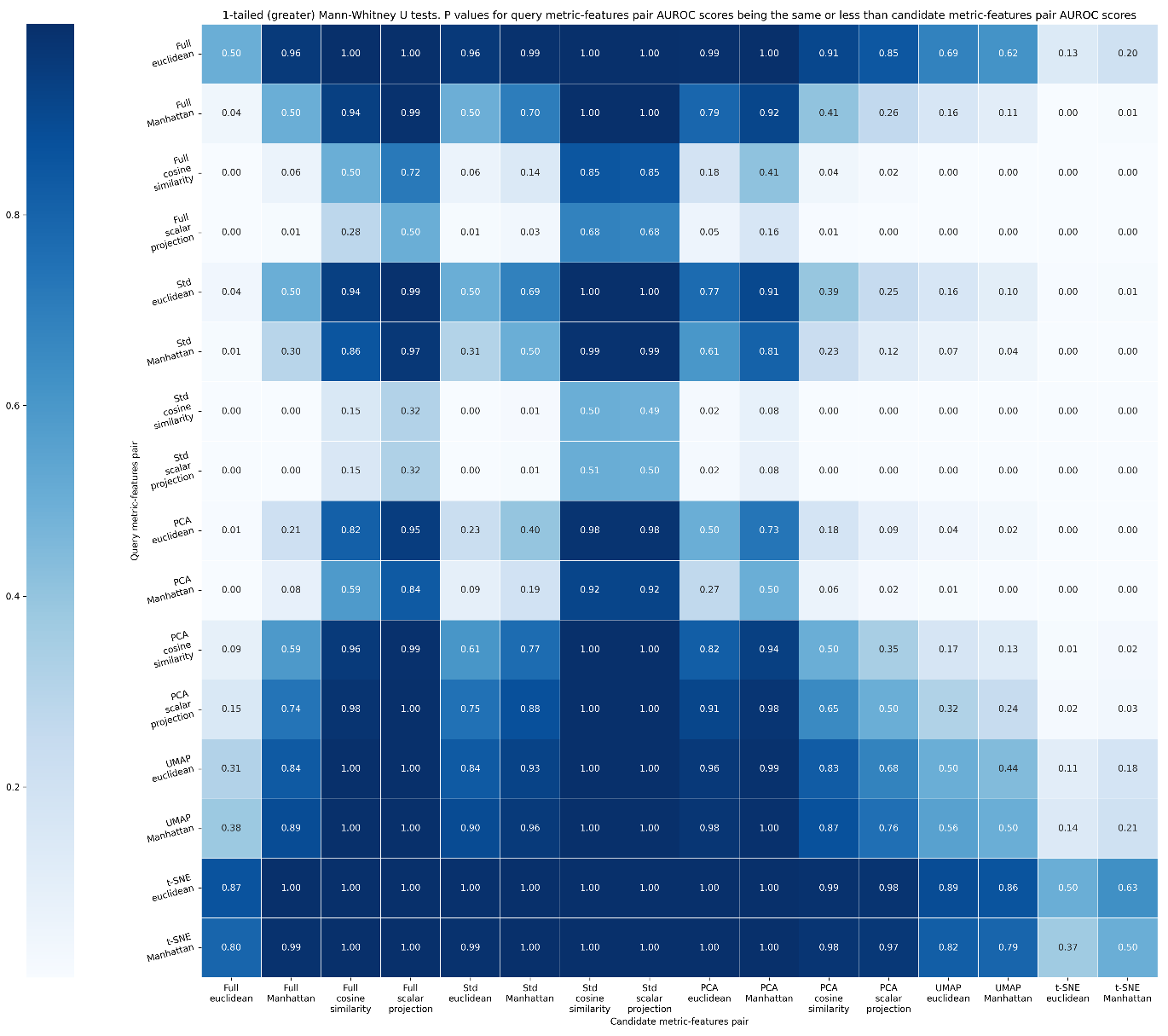


Figure S14 – 1-tailed Mann Whitney-U (greater than) test, evaluating if the query metric-feature space combination is better than the candidate-feature space combination, dimensionality reduction techniques using optimum number of dimensions to reflect theoretical maximum performance. Values denote p-values that the metric-feature space combination is a given row is performed better than the metric-feature space column given in the column by chance along. If this value is <0.05, we deem there to be a less than 5 % chance that the metric-feature space combination outperformed the other by chance.


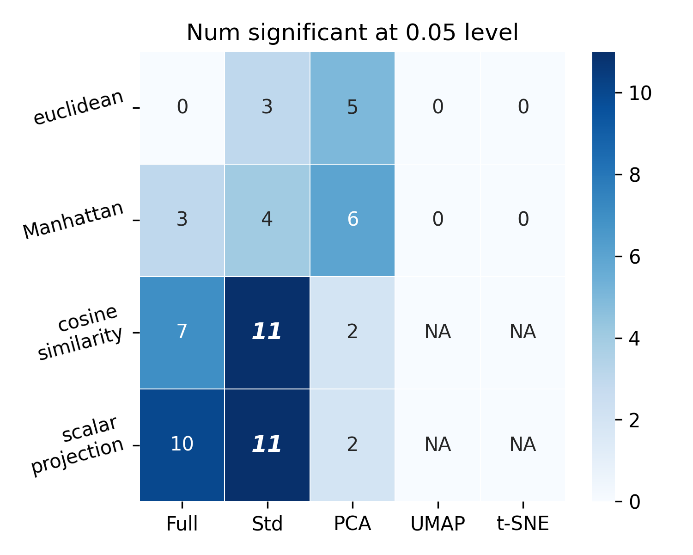


Figure S15 - Counts of the number of features-space metric pairs that were significantly better performing than other metric-feature space pairs. Suggests that scalar projection applied to full and standard scalar feature space outperforms other metrics, however Figure S11 shows there is not a significant performance gain (0.05 level) in using the scalar projection over cosine similarity for full and standard scalar feature spaces.

# References

Lamb, Justin, et al. "The Connectivity Map: using gene-expression signatures to connect small molecules, genes, and disease." science 313.5795 (2006): 1929-1935.

Lee, Changhee, and Mihaela van der Schaar. "A variational information bottleneck approach to multi-omics data integration." International Conference on Artificial Intelligence and Statistics. PMLR, 2021.

Weinstein, John N., et al. "The cancer genome atlas pan-cancer analysis project." Nature genetics 45.10 (2013): 1113-1120.

Wisconsin, <https://archive.ics.uci.edu/ml/datasets/Breast+Cancer+Wisconsin+(Diagnostic)>, (visited 27/4/2022).

Yann, http://yann.lecun.com/exdb/mnist/, (visited 27/4/2022).
